# Crystal structure of 1′-(prop-2-yn-1-yl)-1,4-di­hydro­spiro­[benzo[*d*][1,3]oxazine-2,3′-indolin]-2′-one

**DOI:** 10.1107/S2056989015011949

**Published:** 2015-06-27

**Authors:** Y. AaminaNaaz, J. Kamalraja, G. Vimala, P. T. Perumal, A. SubbiahPandi

**Affiliations:** a Department of Physics, Presidency College (Autonomous), Chennai 600 005, India; b Organic Chemistry Division, Central Leather Research Institute, Adyar, Chennai 602 020, India

**Keywords:** crystal structure, spiro compounds, spiro­oxazines, oxazine, indoline, N—H⋯O hydrogen bonding

## Abstract

In the title compound, C_18_H_14_N_2_O_2_, the six-membered oxazine ring adopts a half-chair conformation and its mean plane makes a dihedral angle of 83.23 (7)° with the pyrrolidine ring of the indoline ring system. In the crystal, mol­ecules are linked *via* N—H⋯O hydrogen bonds, forming chains along [100]. The chains are linked by C—H⋯π inter­actions, forming slabs parallel to (001).

## Related literature   

For the biological activity of spiro compounds, see: James *et al.* (1991 ▸); Kobayashi *et al.* (1991 ▸). For the use of 1,3-dipolar cyclo­addition reactions in the construction of spiro compounds, see: Caramella & Grunanger (1984 ▸). For applications of spiro­oxazine derivatives, see: Chibisov & Görner (1999 ▸). For the synthetic method, see: Kamalraja *et al.* (2014 ▸).
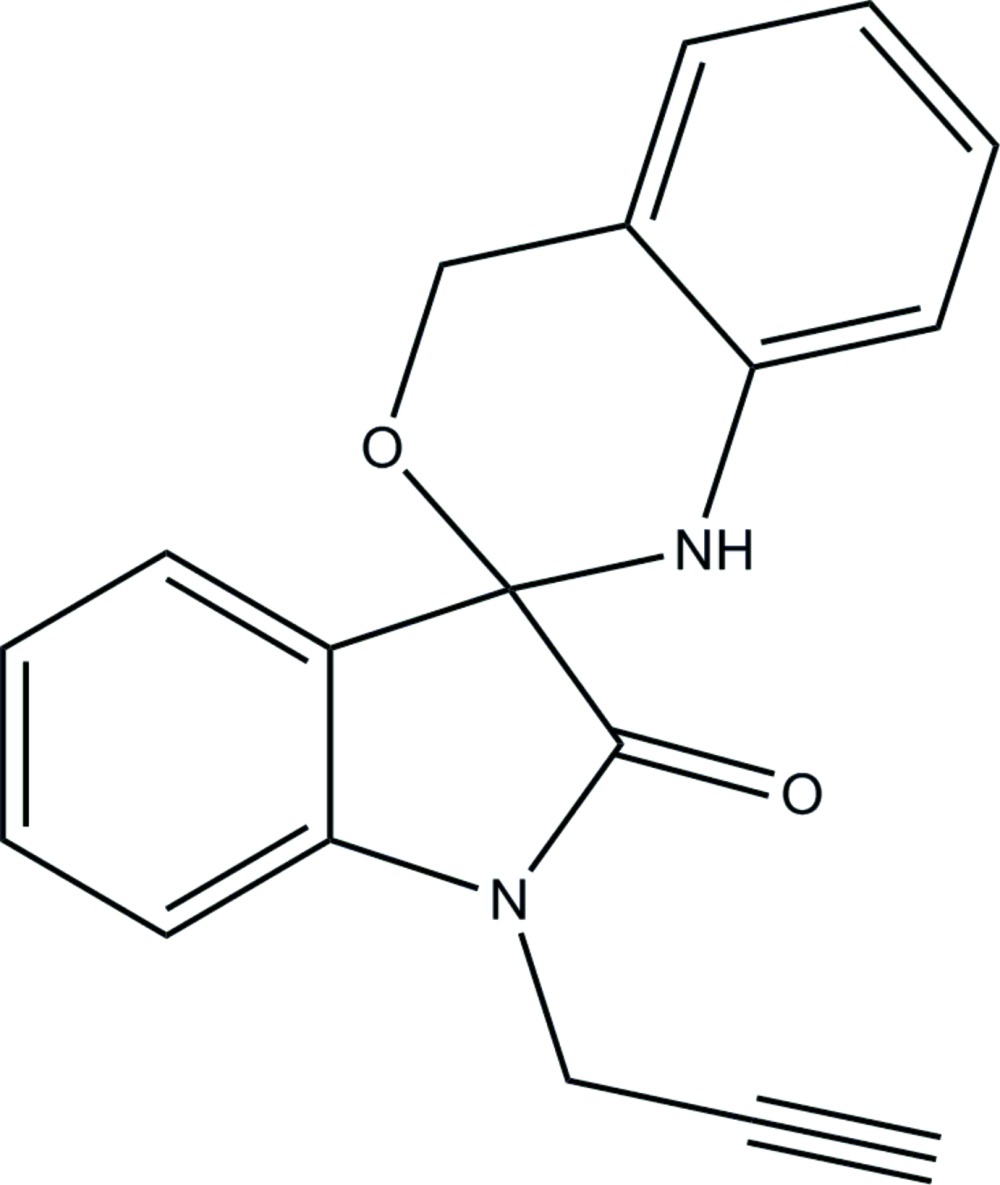


## Experimental   

### Crystal data  

C_18_H_14_N_2_O_2_
*M* *_r_* = 290.31
Triclinic, 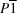

*a* = 5.5571 (3) Å
*b* = 8.5404 (4) Å
*c* = 15.4542 (9) Å
α = 85.884 (3)°
β = 86.814 (3)°
γ = 74.125 (3)°
*V* = 703.17 (6) Å^3^
*Z* = 2
Mo *K*α radiation
μ = 0.09 mm^−1^
*T* = 293 K
0.21 × 0.19 × 0.18 mm

### Data collection  

Bruker SMART APEXII CCD diffractometer
Absorption correction: multi-scan (*SADABS*; Bruker, 2008 ▸) *T*_min_ = 0.981, *T*_max_ = 0.984
16184 measured reflections
3231 independent reflections
2350 reflections with *I* > 2σ(*I*)
*R*_int_ = 0.031

### Refinement  

*R*[*F* ^2^ > 2σ(*F* ^2^)] = 0.041
*wR*(*F* ^2^) = 0.106
*S* = 1.06
3231 reflections
199 parameters
H-atom parameters constrained
Δρ_max_ = 0.15 e Å^−3^
Δρ_min_ = −0.21 e Å^−3^

### 

Data collection: *APEX2* (Bruker, 2008 ▸); cell refinement: *SAINT* (Bruker, 2008 ▸); data reduction: *SAINT*; program(s) used to solve structure: *SHELXS97* (Sheldrick, 2008 ▸); program(s) used to refine structure: *SHELXL97* (Sheldrick, 2008 ▸); molecular graphics: *PLATON* (Spek, 2009 ▸); software used to prepare material for publication: *SHELXL97* and *PLATON*.

## Supplementary Material

Crystal structure: contains datablock(s) global, I. DOI: 10.1107/S2056989015011949/su5155sup1.cif

Structure factors: contains datablock(s) I. DOI: 10.1107/S2056989015011949/su5155Isup2.hkl

Supporting information file. DOI: 10.1107/S2056989015011949/su5155Isup3.cml

**Fig. 1.**
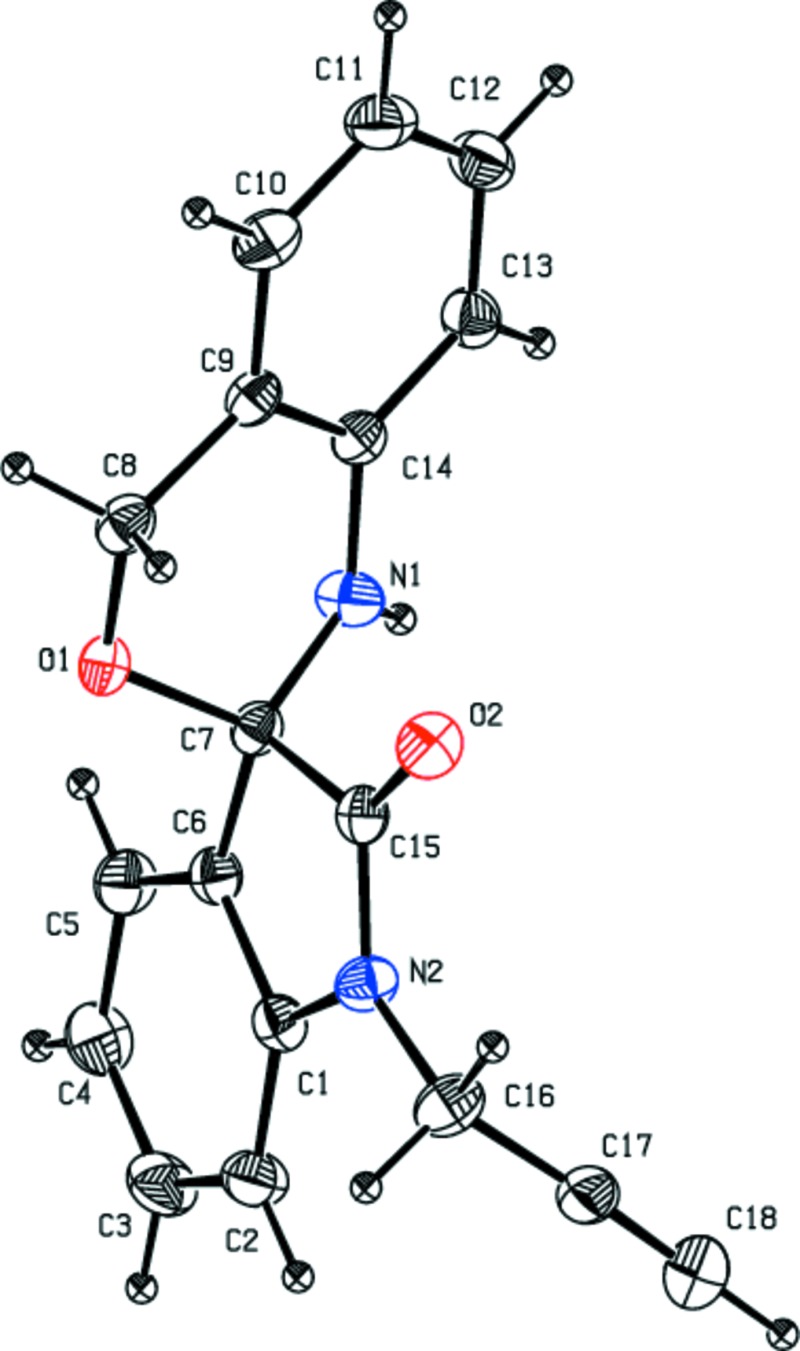
The molecular structure of the title compound, with atom labelling. Displacement ellipsoids are drawn at the 30% probability level.

**Fig. 2.**
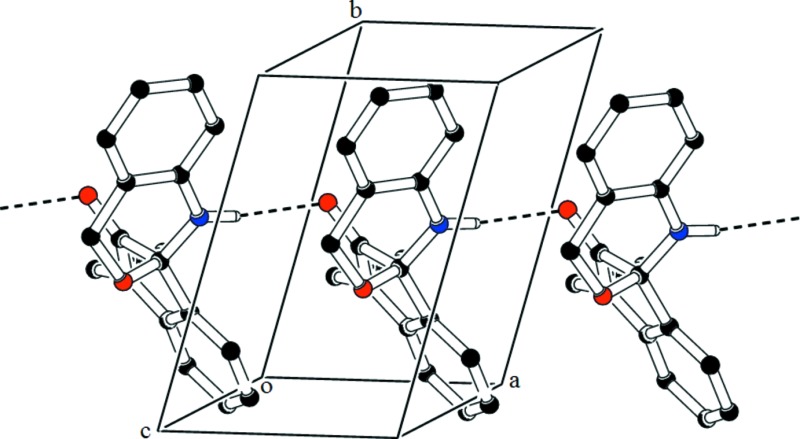
The crystal packing of the title compound, viewed along the c axis. Hydrogen bonds are shown as dashed lines (see Table 1 for details).

. DOI: 10.1107/S2056989015011949/su5155fig1.tif
The mol­ecular structure of the title compound, with atom labelling. Displacement ellipsoids are drawn at the 30% probability level.

c . DOI: 10.1107/S2056989015011949/su5155fig2.tif
The crystal packing of the title compound, viewed along the *c* axis. Hydrogen bonds are shown as dashed lines (see Table 1 for details).

CCDC reference: 1408024

Additional supporting information:  crystallographic information; 3D view; checkCIF report

## Figures and Tables

**Table 1:** Hydrogen-bond geometry (, ) *Cg*3 and *Cg*4 are the centroids of rings C1C6 and C9C14, respectively.

*D*H*A*	*D*H	H*A*	*D* *A*	*D*H*A*
N1H1O2^i^	0.86	2.13	2.9641(16)	164
C4H4*Cg*4^ii^	0.93	2.90	3.6572(19)	140
C8H8*A* *Cg*4^iii^	0.97	2.86	3.6636(17)	141
C16H16*B* *Cg*3^iv^	0.97	2.79	3.5341(18)	134

Symmetry codes: (i) 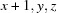; (ii) 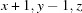; (iii) 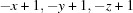; (iv) 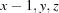.

## supplementary crystallographic information

### S1. Synthesis and crystallization

A mixture of N-propargylisatin (1.0 mmol), and 2-amino­benzyl­alcohol (1.0 mmol) was refluxed in ethanol, in the presence of InCl_3_ (10 mol%), for 2 h. After the reaction was complete as indicated by TLC, the reaction mixture was cooled to room temperature. The solid that formed was filtered, dried and recrystallized in ethanol or di­chloro­methane to obtain in good yield (89%) of the pure title product as block-like colourless crystals.

### S2. Refinement

Crystal data, data collection and structure refinement details are summarized in Table 2. The N- and C-bound H atoms were positioned geometrically (N—H = 0.86 Å, C–H = 0.93–0.97 Å) and allowed to ride on their parent atoms, with *U*_iso_(H) = 1.2*U*_eq_(N,C).

### S3. Structural commentary

Spiro compounds represent an important class of naturally occurring substances, which in many cases exhibit useful biological properties (Kobayashi *et al.*, 1991; James *et al.*, 1991). 1,3-dipolar cyclo­addition reactions are widely used for construction of spiro-compounds (Caramella & Grunanger, 1984). It has also been reported that spiro-oxazine derivatives have real or potential applications in many fields such as protection, decoration, display, memory, switches, photography, photometry and photomechanics (Chibisov & Görner, 1999). Efforts have been made to design this industrially and biologically active hetrocyclic compounds by making or breaking carbon-carbon (C—C) and carbon-hetero atom (C—*X*) (Kamalraja *et al.*, 2014). This InCl_3_-mediated compound have been synthesized as a part of the effort carried to develop eco-friendly potential compound by new synthetic method.

The molecular structure of the title compound is illustrated in Fig 1. The oxazine ring (O1/N1/C7/C8/C9/C14) adopts a half chair confirmation, and its mean plane makes a dihedral angle of 83.23 (7) ° with the pyrrolidine ring (O1/N1/C8/C9/C14) of the indolinone ring system. The indole ring system is essentially planar, with atoms C16 and O2 deviating from its mean plane by -0.0130 and 0.0273 Å, respectively. The dihedral angle between the benzene ring (C1—C6) of the indoline ring system and the benzene ring (C9—C14) of the mean plane of the 2,4-di­hydro-1*H*-benzo[*d*][1,3] oxazine ring system is 76.94 (8) °.

In the crystal, molecules are linked *via* N—H···O hydrogen bonds (Table 1) forming chains along [100], as shown in Fig 2. The chains are linked by C—H···π inter­actions forming slabs parallel to (001); see Table 1.

### Crystal data

**Table:**

C_18_H_14_N_2_O_2_	*Z* = 2
*M**_r_* = 290.31	*F*(000) = 304
Triclinic, *P*1	*D*_x_ = 1.371 Mg m^−^^3^
Hall symbol: -P 1	Mo *K*α radiation, λ = 0.71073 Å
*a* = 5.5571 (3) Å	Cell parameters from 2350 reflections
*b* = 8.5404 (4) Å	θ = 2.5–27.6°
*c* = 15.4542 (9) Å	µ = 0.09 mm^−^^1^
α = 85.884 (3)°	*T* = 293 K
β = 86.814 (3)°	Block, colourless
γ = 74.125 (3)°	0.21 × 0.19 × 0.18 mm
*V* = 703.17 (6) Å^3^	

### Data collection

**Table:**

Bruker SMART APEXII CCD diffractometer	3231 independent reflections
Radiation source: fine-focus sealed tube	2350 reflections with *I* > 2σ(*I*)
Graphite monochromator	*R*_int_ = 0.031
ω and φ scans	θ_max_ = 27.6°, θ_min_ = 2.5°
Absorption correction: multi-scan (*SADABS*; Bruker, 2008)	*h* = −7→7
*T*_min_ = 0.981, *T*_max_ = 0.984	*k* = −11→11
16184 measured reflections	*l* = −20→20

### Refinement

**Table:**

Refinement on *F*^2^	Primary atom site location: structure-invariant direct methods
Least-squares matrix: full	Secondary atom site location: difference Fourier map
*R*[*F*^2^ > 2σ(*F*^2^)] = 0.041	Hydrogen site location: inferred from neighbouring sites
*wR*(*F*^2^) = 0.106	H-atom parameters constrained
*S* = 1.06	*w* = 1/[σ^2^(*F*_o_^2^) + (0.0405*P*)^2^ + 0.1883*P*] where *P* = (*F*_o_^2^ + 2*F*_c_^2^)/3
3231 reflections	(Δ/σ)_max_ < 0.001
199 parameters	Δρ_max_ = 0.15 e Å^−^^3^
0 restraints	Δρ_min_ = −0.21 e Å^−^^3^

### Special details

**Table:**

Geometry. All e.s.d.'s (except the e.s.d. in the dihedral angle between two l.s. planes) are estimated using the full covariance matrix. The cell e.s.d.'s are taken into account individually in the estimation of e.s.d.'s in distances, angles and torsion angles; correlations between e.s.d.'s in cell parameters are only used when they are defined by crystal symmetry. An approximate (isotropic) treatment of cell e.s.d.'s is used for estimating e.s.d.'s involving l.s. planes.
Refinement. Refinement of *F*^2^ against ALL reflections. The weighted *R*-factor *wR* and goodness of fit *S* are based on *F*^2^, conventional *R*-factors *R* are based on *F*, with *F* set to zero for negative *F*^2^. The threshold expression of *F*^2^ > σ(*F*^2^) is used only for calculating *R*-factors(gt) *etc*. and is not relevant to the choice of reflections for refinement. *R*-factors based on *F*^2^ are statistically about twice as large as those based on *F*, and *R*- factors based on ALL data will be even larger.

### Fractional atomic coordinates and isotropic or equivalent isotropic displacement parameters (Å^2^)

**Table:**

	*x*	*y*	*z*	*U*_iso_*/*U*_eq_	
O1	0.44655 (19)	0.31187 (12)	0.35868 (6)	0.0365 (3)	
N1	0.6584 (2)	0.48707 (15)	0.28492 (8)	0.0383 (3)	
H1	0.7953	0.4869	0.2560	0.046*	
O2	0.14706 (18)	0.52580 (13)	0.21423 (7)	0.0413 (3)	
N2	0.3795 (2)	0.29626 (15)	0.15105 (8)	0.0352 (3)	
C6	0.7141 (3)	0.21178 (17)	0.23854 (9)	0.0322 (3)	
C9	0.3552 (3)	0.59601 (17)	0.39673 (9)	0.0321 (3)	
C14	0.5492 (2)	0.61191 (17)	0.33911 (9)	0.0299 (3)	
C7	0.5434 (2)	0.35940 (17)	0.27729 (9)	0.0296 (3)	
C13	0.6337 (3)	0.75102 (18)	0.33677 (10)	0.0379 (3)	
H13	0.7673	0.7600	0.2996	0.045*	
C15	0.3291 (2)	0.40839 (17)	0.21203 (9)	0.0306 (3)	
C8	0.2756 (3)	0.44185 (18)	0.40275 (10)	0.0369 (3)	
H8A	0.2598	0.4071	0.4635	0.044*	
H8B	0.1120	0.4635	0.3782	0.044*	
C10	0.2424 (3)	0.7231 (2)	0.44848 (10)	0.0428 (4)	
H10	0.1106	0.7142	0.4865	0.051*	
C1	0.6078 (3)	0.17761 (17)	0.16557 (9)	0.0329 (3)	
C12	0.5198 (3)	0.87512 (19)	0.38939 (11)	0.0453 (4)	
H12	0.5767	0.9681	0.3877	0.054*	
C17	0.3297 (3)	0.31873 (19)	−0.00454 (11)	0.0432 (4)	
C16	0.2147 (3)	0.2998 (2)	0.08103 (10)	0.0442 (4)	
H16A	0.0651	0.3894	0.0879	0.053*	
H16B	0.1641	0.1994	0.0851	0.053*	
C5	0.9381 (3)	0.11227 (19)	0.26579 (10)	0.0420 (4)	
H5	1.0113	0.1349	0.3144	0.050*	
C11	0.3219 (3)	0.8629 (2)	0.44470 (11)	0.0481 (4)	
H11	0.2425	0.9483	0.4792	0.058*	
C3	0.9441 (3)	−0.05574 (19)	0.14768 (12)	0.0501 (4)	
H3	1.0233	−0.1477	0.1177	0.060*	
C2	0.7189 (3)	0.04466 (19)	0.11901 (11)	0.0438 (4)	
H2	0.6462	0.0227	0.0701	0.053*	
C4	1.0533 (3)	−0.0227 (2)	0.21942 (12)	0.0496 (4)	
H4	1.2056	−0.0915	0.2369	0.060*	
C18	0.4183 (4)	0.3288 (2)	−0.07400 (13)	0.0641 (5)	
H18	0.4891	0.3368	−0.1295	0.077*	

### Atomic displacement parameters (Å^2^)

**Table:**

	*U*^11^	*U*^22^	*U*^33^	*U*^12^	*U*^13^	*U*^23^
O1	0.0434 (6)	0.0375 (5)	0.0296 (5)	−0.0140 (5)	0.0021 (4)	0.0025 (4)
N1	0.0319 (6)	0.0435 (7)	0.0449 (7)	−0.0192 (6)	0.0108 (5)	−0.0129 (6)
O2	0.0295 (5)	0.0451 (6)	0.0435 (6)	0.0000 (5)	−0.0013 (4)	−0.0031 (5)
N2	0.0304 (6)	0.0400 (7)	0.0345 (7)	−0.0064 (5)	−0.0075 (5)	−0.0056 (5)
C6	0.0310 (7)	0.0313 (7)	0.0336 (7)	−0.0080 (6)	−0.0025 (6)	0.0011 (6)
C9	0.0294 (7)	0.0403 (8)	0.0267 (7)	−0.0093 (6)	−0.0037 (6)	−0.0011 (6)
C14	0.0263 (7)	0.0343 (7)	0.0294 (7)	−0.0078 (6)	−0.0055 (5)	−0.0013 (6)
C7	0.0286 (7)	0.0343 (7)	0.0270 (7)	−0.0106 (6)	−0.0007 (5)	−0.0003 (6)
C13	0.0370 (8)	0.0390 (8)	0.0406 (8)	−0.0154 (7)	−0.0039 (6)	−0.0001 (7)
C15	0.0260 (7)	0.0342 (7)	0.0321 (7)	−0.0102 (6)	0.0018 (6)	0.0012 (6)
C8	0.0350 (8)	0.0468 (9)	0.0310 (7)	−0.0155 (7)	0.0041 (6)	−0.0023 (6)
C10	0.0388 (9)	0.0531 (9)	0.0352 (8)	−0.0096 (7)	0.0022 (7)	−0.0084 (7)
C1	0.0317 (7)	0.0305 (7)	0.0365 (8)	−0.0085 (6)	−0.0032 (6)	0.0002 (6)
C12	0.0525 (10)	0.0362 (8)	0.0502 (10)	−0.0149 (7)	−0.0113 (8)	−0.0037 (7)
C17	0.0513 (10)	0.0395 (8)	0.0392 (9)	−0.0107 (7)	−0.0122 (7)	−0.0028 (7)
C16	0.0373 (8)	0.0585 (10)	0.0390 (9)	−0.0140 (7)	−0.0111 (7)	−0.0057 (7)
C5	0.0376 (8)	0.0429 (9)	0.0426 (9)	−0.0054 (7)	−0.0098 (7)	0.0008 (7)
C11	0.0531 (10)	0.0440 (9)	0.0449 (9)	−0.0060 (8)	−0.0048 (8)	−0.0142 (7)
C3	0.0519 (10)	0.0332 (8)	0.0597 (11)	−0.0012 (7)	0.0001 (8)	−0.0083 (8)
C2	0.0480 (9)	0.0363 (8)	0.0469 (9)	−0.0084 (7)	−0.0061 (7)	−0.0087 (7)
C4	0.0412 (9)	0.0395 (9)	0.0596 (11)	0.0032 (7)	−0.0066 (8)	0.0013 (8)
C18	0.0887 (15)	0.0581 (12)	0.0450 (11)	−0.0205 (11)	−0.0025 (10)	0.0033 (9)

### Geometric parameters (Å, º)

**Table:**

O1—C7	1.4168 (16)	C9—C8	1.495 (2)
O1—C8	1.4347 (17)	C14—C13	1.390 (2)
N1—C14	1.3872 (17)	C7—C15	1.5525 (19)
N1—C7	1.4212 (17)	C13—C12	1.373 (2)
O2—C15	1.2150 (16)	C10—C11	1.378 (2)
N2—C15	1.3554 (18)	C1—C2	1.368 (2)
N2—C1	1.4078 (18)	C12—C11	1.377 (2)
N2—C16	1.4497 (18)	C17—C18	1.163 (2)
C6—C5	1.370 (2)	C17—C16	1.454 (2)
C6—C1	1.3861 (19)	C5—C4	1.384 (2)
C6—C7	1.4964 (19)	C3—C4	1.375 (2)
C9—C10	1.381 (2)	C3—C2	1.385 (2)
C9—C14	1.3888 (19)		
			
C7—O1—C8	114.81 (10)	C6—C7—C15	101.78 (11)
C14—N1—C7	119.77 (11)	C12—C13—C14	119.93 (15)
C15—N2—C1	111.34 (11)	O2—C15—N2	125.26 (13)
C15—N2—C16	123.33 (12)	O2—C15—C7	126.81 (13)
C1—N2—C16	125.32 (12)	N2—C15—C7	107.93 (11)
C5—C6—C1	120.23 (13)	O1—C8—C9	113.31 (11)
C5—C6—C7	130.43 (13)	C11—C10—C9	121.15 (15)
C1—C6—C7	109.30 (12)	C2—C1—C6	121.96 (14)
C10—C9—C14	118.87 (14)	C2—C1—N2	128.46 (13)
C10—C9—C8	121.35 (13)	C6—C1—N2	109.58 (12)
C14—C9—C8	119.77 (12)	C13—C12—C11	120.49 (15)
N1—C14—C9	119.32 (12)	C18—C17—C16	177.26 (18)
N1—C14—C13	120.65 (13)	N2—C16—C17	113.13 (13)
C9—C14—C13	120.03 (13)	C6—C5—C4	118.59 (15)
O1—C7—N1	111.37 (11)	C12—C11—C10	119.46 (15)
O1—C7—C6	108.82 (11)	C4—C3—C2	121.54 (15)
N1—C7—C6	113.52 (11)	C1—C2—C3	117.24 (15)
O1—C7—C15	108.92 (10)	C3—C4—C5	120.44 (15)
N1—C7—C15	111.96 (11)		
			
C7—N1—C14—C9	11.5 (2)	N1—C7—C15—N2	124.05 (12)
C7—N1—C14—C13	−169.20 (13)	C6—C7—C15—N2	2.48 (14)
C10—C9—C14—N1	−177.99 (13)	C7—O1—C8—C9	−41.18 (16)
C8—C9—C14—N1	3.0 (2)	C10—C9—C8—O1	−167.24 (13)
C10—C9—C14—C13	2.7 (2)	C14—C9—C8—O1	11.71 (19)
C8—C9—C14—C13	−176.28 (13)	C14—C9—C10—C11	−1.1 (2)
C8—O1—C7—N1	55.09 (15)	C8—C9—C10—C11	177.88 (14)
C8—O1—C7—C6	−179.03 (11)	C5—C6—C1—C2	0.6 (2)
C8—O1—C7—C15	−68.86 (14)	C7—C6—C1—C2	−177.30 (13)
C14—N1—C7—O1	−39.97 (17)	C5—C6—C1—N2	179.83 (13)
C14—N1—C7—C6	−163.21 (12)	C7—C6—C1—N2	1.91 (16)
C14—N1—C7—C15	82.24 (15)	C15—N2—C1—C2	178.94 (15)
C5—C6—C7—O1	−65.35 (19)	C16—N2—C1—C2	−0.4 (2)
C1—C6—C7—O1	112.28 (13)	C15—N2—C1—C6	−0.21 (16)
C5—C6—C7—N1	59.3 (2)	C16—N2—C1—C6	−179.52 (14)
C1—C6—C7—N1	−123.10 (13)	C14—C13—C12—C11	0.0 (2)
C5—C6—C7—C15	179.75 (15)	C15—N2—C16—C17	118.22 (16)
C1—C6—C7—C15	−2.62 (14)	C1—N2—C16—C17	−62.5 (2)
N1—C14—C13—C12	178.50 (13)	C18—C17—C16—N2	133 (4)
C9—C14—C13—C12	−2.2 (2)	C1—C6—C5—C4	−0.5 (2)
C1—N2—C15—O2	178.55 (13)	C7—C6—C5—C4	176.88 (15)
C16—N2—C15—O2	−2.1 (2)	C13—C12—C11—C10	1.6 (2)
C1—N2—C15—C7	−1.51 (15)	C9—C10—C11—C12	−1.1 (2)
C16—N2—C15—C7	177.82 (13)	C6—C1—C2—C3	0.0 (2)
O1—C7—C15—O2	67.59 (17)	N2—C1—C2—C3	−179.08 (15)
N1—C7—C15—O2	−56.01 (18)	C4—C3—C2—C1	−0.6 (3)
C6—C7—C15—O2	−177.58 (13)	C2—C3—C4—C5	0.7 (3)
O1—C7—C15—N2	−112.35 (12)	C6—C5—C4—C3	−0.1 (3)

### Hydrogen-bond geometry (Å, º)

Cg3 and Cg4 are the centroids of rings C1–C6 and C9–C14, respectively.

**Table:**

*D*—H···*A*	*D*—H	H···*A*	*D*···*A*	*D*—H···*A*
N1—H1···O2^i^	0.86	2.13	2.9641 (16)	164
C4—H4···*Cg*4^ii^	0.93	2.90	3.6572 (19)	140
C8—H8*A*···*Cg*4^iii^	0.97	2.86	3.6636 (17)	141
C16—H16*B*···*Cg*3^iv^	0.97	2.79	3.5341 (18)	134

Symmetry codes: (i) *x*+1, *y*, *z*; (ii) *x*+1, *y*−1, *z*; (iii) −*x*+1, −*y*+1, −*z*+1; (iv) *x*−1, *y*, *z*.
