# Supplementary figures and images for: Crystal structure of 1′-(prop-2-yn-1-yl)-1,4-di­hydro­spiro­[benzo[d][1,3]oxazine-2,3′-indolin]-2′-one
Source: Acta Crystallogr E Crystallogr Commun. 2015 Jun 27;71(Pt 7):o510–1. doi: 10.1107/S2056989015011949 (PMC4518956; doi:10.1107/S2056989015011949)

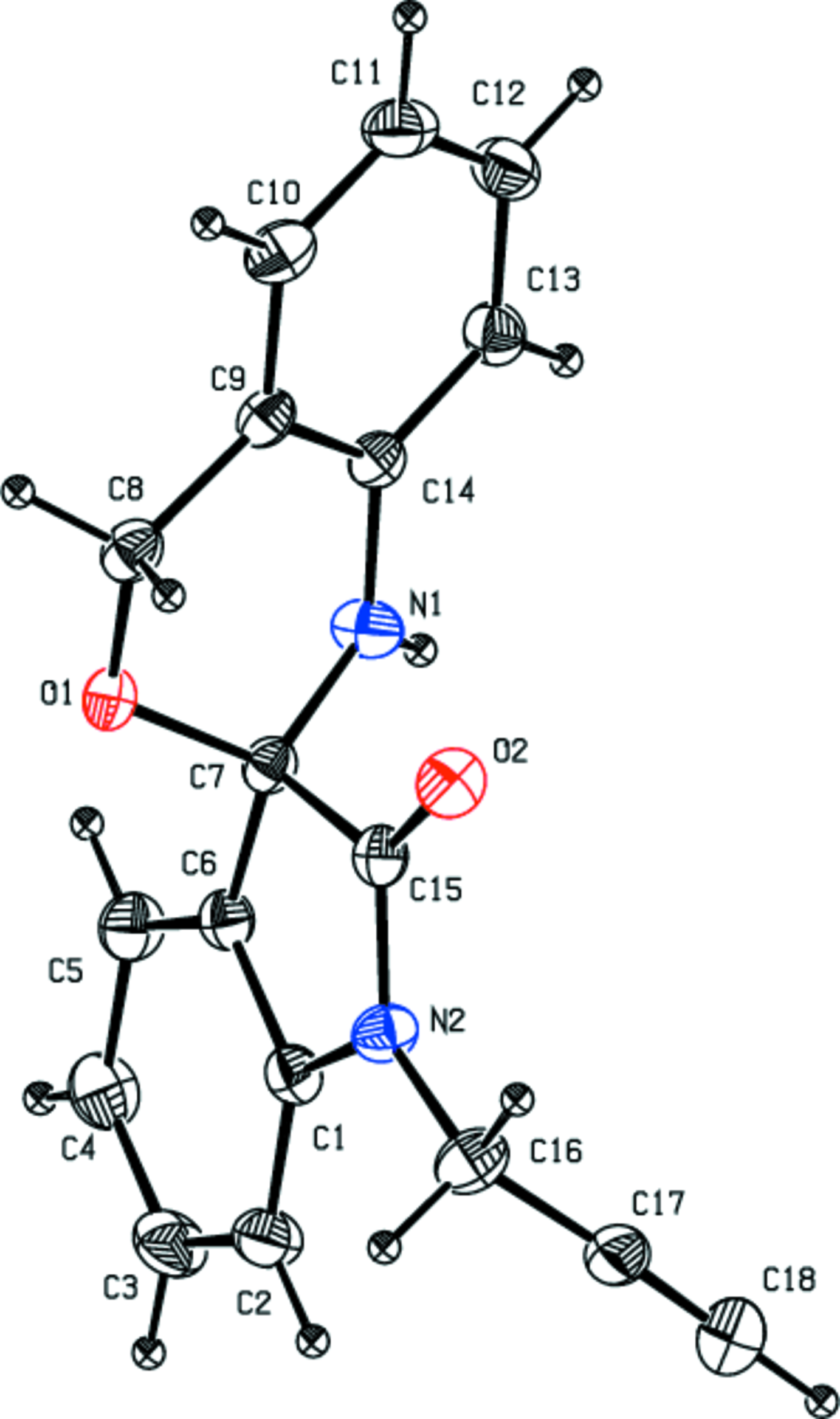

Supplement: Supplementary file 4 [file e-71-0o510-fig1.tif]

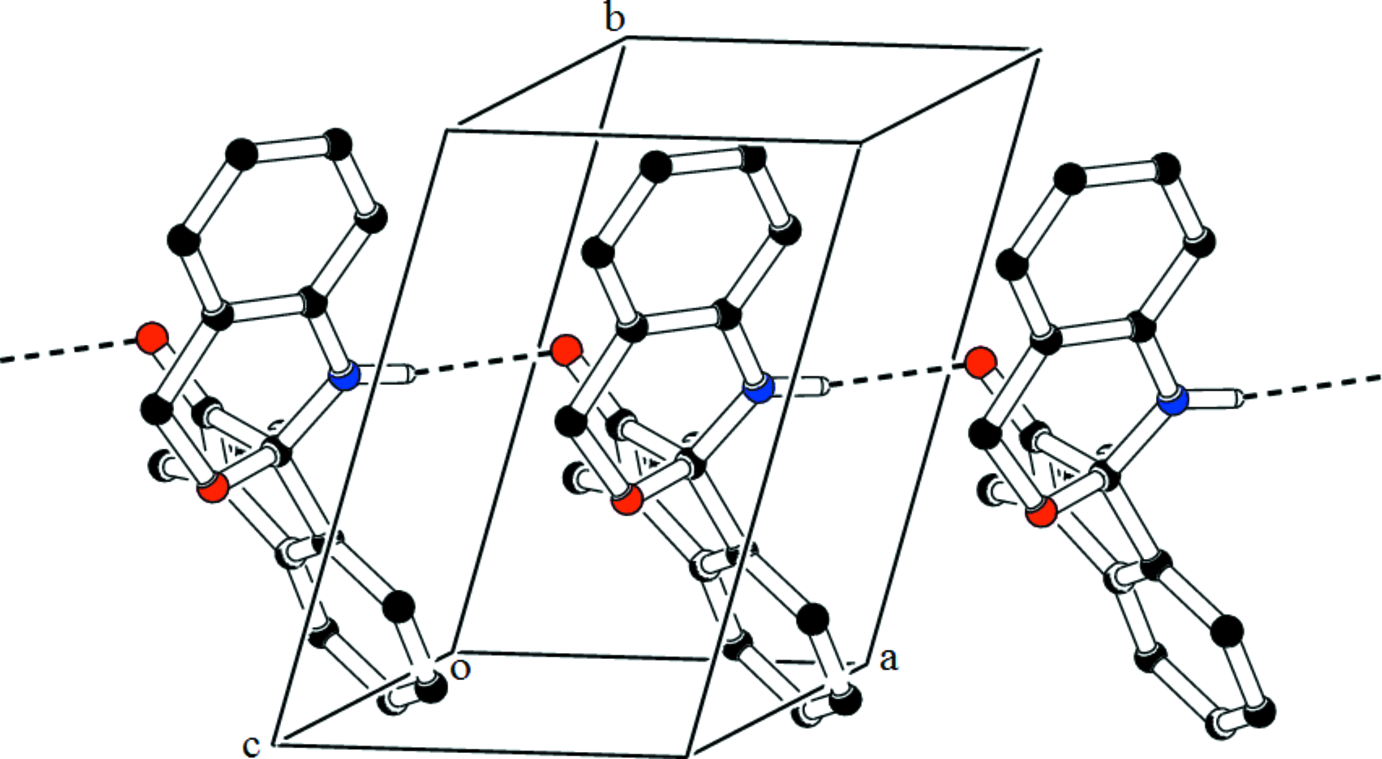

Supplement: Supplementary file 5 [file e-71-0o510-fig2.tif]
